# The Effect of the Supervisor–Student Relationship on Academic Procrastination: The Chain-Mediating Role of Academic Self-Efficacy and Learning Adaptation

**DOI:** 10.3390/ijerph19052621

**Published:** 2022-02-24

**Authors:** Qinglin Wang, Zhaoyang Xin, Hang Zhang, Jing Du, Minghui Wang

**Affiliations:** 1 School of Psychology, Henan University, Kaifeng 475004, China; wqlwang2021@163.com (Q.W.); psyxin2021@163.com (Z.X.); zxcvbnmlp05@163.com (H.Z.); 2 Faculty of Education, Henan University, Kaifeng 475004, China; dujing@henu.edu.cn

**Keywords:** supervisor–student relationship, academic self-efficacy, learning adaptation, academic procrastination, chain-mediating effect

## Abstract

This study used a sample of 818 postgraduate students across several universities in China, to explore the effect of the supervisor–student relationship on procrastination in postgraduates, and the mediating roles played by the postgraduates’ academic self-efficacy and learning adaptation. The study employs multiple scales and finds that: (1) the relationship between postgraduates and their supervisors is significantly and negatively correlated with academic procrastination; (2) the academic self-efficacy of postgraduates plays an independent intermediary role in the connection between the supervisor–student relationship and academic procrastination; (3) the learning adaptation of postgraduates also plays an independent intermediary role in the connection between the supervisor–student relationship and academic procrastination; (4) the academic self-efficacy and learning adaptation of postgraduates shows a chain-mediating effect in the connection between the supervisor–student relationship and academic procrastination. Ultimately, the supervisor–student relationship is an important factor that can directly affect academic procrastination, even if postgraduate students display academic self-efficacy and learning adaptation.

## 1. Introduction

Academic procrastination is defined as intentionally deferring or delaying academic work that must be completed [1]. Studies across the world have found a high incidence of academic procrastination among college students, and some studies have also found that graduate students exhibit high levels of academic procrastination [2,3,4]. Academic procrastination not only produces negative emotions, such as anxiety, which affects one’s mental health, but also has a significant impact on one’s academic performance [5,6,7]. For graduate students, if there is a higher degree of academic procrastination, they not only have to bear the heavy pressure of research, but also have to face the emotional disorder brought about by academic procrastination, which will have a serious negative impact on their physical and mental health. Thus, this paper probes into the influencing factors and mechanisms of academic procrastination in postgraduates, and puts forward some suggestions for mitigating it. Prior research on academic procrastination has tended to focus on the influence of personality traits, time management, motivation levels, and so on [8,9,10]. To date, there has been limited research on the influence of the supervisor–student relationship on academic procrastination, which this study focuses on, including the mediating role of academic self-efficacy and learning adaptation.

The relationship between graduate students and their supervisors is established on the basis of talent cultivation and scientific research, and is developed in the course of daily interactions [11]. This relationship not only affects the student’s creativity and research performance of graduate students, but also has a significant impact on graduate students’ behaviors, such as academic misconduct and learning engagement [11,12,13,14]. In addition, according to the leader–member exchange theory, if a subordinate maintains a higher exchange relationship with the leader characterized by a high degree of trust, communication, support, and loyalty, the leader will regard the subordinate as an “insider” and provide more care, support, and resources to the subordinate. In turn, the subordinate will reduce negative behaviors based on the principle of reciprocity in social exchange, such as procrastination. In a productive relationship between the supervisor and graduate student, the supervisor provides the material, emotional, and cognitive resources for the graduate students [15]. Based on the principle of reciprocity of social exchange, graduate students will complete the academic tasks assigned by their supervisors on time, which will reduce the occurrence of academic procrastination. Therefore, this study proposes Hypothesis 1: the supervisor–student relationship between graduate students and supervisors negatively affects graduate students’ academic procrastination.

Academic self-efficacy refers to an individual’s belief in his/her ability to perform academic research tasks [16,17]. According to Bandura’s self-efficacy theory, the self-efficacy of individuals will have an impact on their behavior, on their choice of behavior in the face of difficulties, and on the persistence of their behavior [18]. People with high self-efficacy tend to adapt and change their environment proactively, work hard to overcome difficulties, and persist for longer periods of time. Therefore, when individuals have high academic self-efficacy, even if the research tasks assigned by supervisors are difficult, graduate students will actively think of solutions and strive to complete the research tasks on time, thus reducing the occurrence of academic procrastination. Previous studies have found that academic self-efficacy can negatively predict academic procrastination [19,20,21]. For example, Li found that the academic self-efficacy of postgraduates significantly predicted their levels of procrastination [22]. Social cognitive theory holds that self-efficacy is influenced by the expectations, guidance, and social support of important people in one’s life, and good interpersonal relationships promote the development of self-efficacy [23,24,25,26]. Therefore, a good relationship characterized by a high degree of trust, communication, and support between graduate students and their supervisors can help the student to obtain more support and resources from their supervisor, which results in higher academic self-efficacy. The research of Chu et al. found that the supervisor–student relationship could significantly predict the self-efficacy of postgraduates [27]. A follow-up study found that the presence or absence of a mentor at the beginning of the semester influenced the self-efficacy of college students at the end of the semester [28]. Yao and Yu also found that the support of one’s supervisor could positively predict the academic self-efficacy of graduate students, and that the effect of the supervisor’s support on academic self-efficacy is more pronounced under the condition of high peer support [19]. Ren found the supervisor support had a positive effect on the academic self-efficacy of graduate students in research [29]. Thus, this study contends that when graduate students have a good supervisor–student relationship, they can reach a higher degree of academic self-efficacy, and thus reduce academic procrastination. In other words, the academic self-efficacy of postgraduate students mediates the relationship between the supervisor–student relationship and academic procrastination. In addition, research has found that the academic self-efficacy of graduate students mediates the following relationships: between an orientation towards goal achievement and academic procrastination [20]; between mobile phone dependence and academic procrastination [30]; and the capacity for perfectionism to predict academic procrastination [31]. This leads to Hypothesis 2 of this study: academic self-efficacy mediates the connection between the supervisor–student relationship and academic procrastination.

Learning adaptation is a behavioral process in which subjects try to adjust themselves to balance their learning circumstances according to their circumstances and learning needs [32]. According to Jean Piaget’s cognitive equilibrium theory, adaptation is a state of equilibrium that individuals achieve through assimilation and accommodation [33,34]. Compared with the undergraduate course, engaging in postgraduate study involves a fundamental change in the content of study and the goal of study, which is more challenging and more demanding, there is thus also a need to take advantage of assimilation and accommodation to incorporate new information and to adjust their knowledge structures, which is oriented to achieve the state of adaptation. According to Bronfenbrenner’s developing ecology theory, the process of an individual adapting to a new environment is affected by external environmental factors which occur at four levels: micro, meso, external, and macro [35,36]. The micro- and meso-environment in which the graduate student operates has an important impact. As one factor of the meso-environment, the supervisor–student relationship can affect the adaptation of graduate students. If graduate students have a good relationship with their supervisor, they can obtain the support and help they require to achieve adaptability. Research has also found that the mode of instruction, the relationship with the supervisor, and the care and help of the supervisor can influence the learning adaptation of graduate students [37,38]. When graduate students are well adapted to learning, they have sufficient resources to cope with the difficulties that arise in the learning process and are able to find solutions in the face of challenging academic tasks. Studies have also found that learning adaptation can significantly predict learning procrastination, and further influence academic achievement through academic procrastination [39]. Therefore, this study proposes Hypothesis 3: learning adaptation plays a mediating role between the supervisor–student relationship and academic procrastination.

Furthermore, according to Bandura’s self-efficacy theory, individuals with high self-efficacy are able to actively adapt and change their environment, even in the face of significant changes in learning styles and learning content. Individuals with high self-efficacy are capable of finding ways to overcome difficulties, adapt to new environments, and have better learning adaptation. Studies involving junior high school students and postgraduate students have also found that academic self-efficacy can significantly predict learning adaptation [40,41]. For graduate students, the main learning task during the study period is to complete the individually driven, lengthy pieces of research tasks on time. This study contends that if graduate students have a good relationship with their supervisor, which in turns product higher self-efficacy in academic research, they can adapt better to the scientific research environment and have sufficient time and resources to overcome the challenges that arise in postgraduate study. Hypothesis 4 is as follows: academic self-efficacy and learning adaptation play a chain-mediated role between the supervisor–student relationship and academic procrastination.

To sum up, this research will start from the relationship between supervisors and students, and take the graduate students as subjects—based on the theory of leader–member exchange, social cognitive theory, and developing ecology theory—to examine the mediating role of academic self-efficacy and learning adaptation in the relationship between the supervisor–student relationship and academic procrastination, and the chain-mediating role of academic self-efficacy and learning adaptation. The purpose is to clarify the influence of the supervisor–student relationship on academic procrastination, providing theoretical support and insights to mitigate it.

## 2. Materials and Methods

### 2.1. Participants

According to the empirical rule, the sample number is generally 10–20 times the number of variables. Using the convenient sampling method, 818 valid questionnaires were collected from graduate students in Henan province (doctoral students were excluded), according to the arrangement of the graduate college of the universities. There were 228 male students (27.9%), 590 female students (72.1%), comprising 453 first-year graduate students (55.4%), 291 second-year graduate students (35.6%), and 74 third-year graduate students (9.0%). There were 440 (53.8%) graduate students from the Humanities and Social Science area, and 378 (46.2%) graduate students from the Natural Sciences area.

### 2.2. Measures

The supervisor–student relationship was measured using a single-dimension, 7-item questionnaire relating to student perceptions of this relationship (e.g., “I’m sure my supervisor is very satisfied with my current scientific performance”) [11]. The scale adopts a 6-point Likert scoring method with participants selecting ranking the statements from 1 = strongly disagree to 6 = strongly agree. The higher the total score, the better the supervisor–student relationship is perceived to be. The Cronbach’s *α* for this study was 0.934.

Academic self-efficacy was measured using the self-efficacy questionnaire [14], which is single-dimensional and includes three questions (e.g., “I am confident that I can do scientific research”). A 6-point Likert scoring method was also used with high total scores indicating high levels of academic self-efficacy. The Cronbach’s *α* for this study was 0.949.

Learning adaptation was measured using a revised questionnaire for graduate students (e.g., “I feel like I’ve lost my goal of learning”) [42], which comprised 5 dimensions—learning motivation, learning ability, learning attitude, learning environment, and external environment—and 20 items. Again, the scale adopted the 6-point Likert scoring method, with higher total scores indicating higher levels of learning adaptation. The Cronbach’s *α* for this study were 0.859 for learning motivation, 0.807 for learning ability, 0.732 for learning attitude, 0.816 for learning environment, and 0.698 for external environment.

Academic procrastination was measured using the 12-item graduate students’ academic procrastination questionnaire (e.g., “Did you procrastinate on the task”) [43]. The scale adopts a 5-point Likert scoring method with higher scores indicating greater tendencies towards academic procrastination. The Cronbach’s *α* for this study was 0.873.

### 2.3. Data Analysis

We used SPSS 21.0 (IBM Corp., Armonk, NY, USA) to provide descriptive analyses of the variables, including the means, standard deviations, and Pearson correlations, and test the possibility of common method deviation in the survey data. Mplus 8.3 was used to examine the hypothetical model using structural equation model. We used chi-square values (*χ*^2^/*df*), the comparative fit index (CFI), the Tucker–Lewis fit index (TLI), the root mean square error of approximation (RMSEA), and the standardized root mean square residual (SRMR) to evaluate the models. In general, an acceptable model fit is indicated by CFI and TLI greater than 0.9 and RMSEA and SRMR less than 0.08.

## 3. Results

### 3.1. Common Method Bias Test

Because multiple variables in this study are provided by the same people when completing the questionnaire, there is a possibility that our data may suffer from the common method bias effect. Previous research indicates that Harman’s single-factor test is useful to evaluate the influence of common method bias [44]. The measurement items of all the variables in this study were used in a factor analysis, and it was found that there were 6 factors extracted in this study. The variance of the first factor was only 34.124%, less than the critical criterion of 40%, which indicates that common method bias was not a serious issue in this study.

### 3.2. Descriptive Statistics and Correlation Analysis

Table 1 lists the average value, standard deviation, and correlation coefficient matrix of each variable. There is a significant positive correlation between academic self-efficacy and the supervisor–student relationship (*r* = 0.627, *p* < 0.01). There is also a significant positive correlation between learning adaptation and the supervisor–student relationship and academic self-efficacy (*r* = 0.648, *p* < 0.01; *r* = 0.598, *p* < 0.01). Finally, we found a significant negative correlation between academic procrastination and the supervisor–student relationship, academic self-efficacy, and learning adaptation (*r* = −0.399, *p* < 0.01; *r* = −0.424, *p* < 0.01; *r* = −0.491, *p* < 0.01).

### 3.3. Chain-Mediated Effect Test

In line with the mediation effect test, we employed the structural equation model method to further evaluate the chain-mediated effect of academic self-efficacy and learning adaptation between the supervisor–student relationship and academic procrastination [45,46]. Firstly, the effects of the supervisor–student relationship on academic procrastination were examined. The fitting indices of the model were *χ*^2^/*df* = 9.383, CFI = 0.944, TLI = 0.929, and SRMR = 0.034, and the model fitted well. Model 1 showed that the supervisor–student relationship negatively predicted academic procrastination (*β* = −0.438, *p* < 0.001), supporting Hypothesis 1 (see Table 2). Secondly, in Model 2, the variables of academic self-efficacy and learning adaptation were added to Model 1 to test their chain-mediated effects. The fitting indices of Model 2 were *χ*^2^/*df* = 5.295, CFI = 0.949, TLI = 0.941, RMSEA = 0.072, and SRMR = 0.037, indicating that the model fitted well [47,48]. Model 2 showed that the supervisor–student relationship did not predict academic procrastination (*β* = −0.026, *p* > 0.05), academic self-efficacy was a significant and negative predictor of academic procrastination (*β* = −0.125, *p* < 0.05), and learning adaptation significantly negatively predicted academic procrastination (*β* = −0.449, *p* < 0.001) (see Table 2).

The results of our analysis showed that the mediating effect between the supervisor–student relationship and academic procrastination was −0.413, accounting for 94.29% of the total effect (the direct effect was −0.026). The mediating effect includes three paths. In the first path, the mediating effect of academic self-efficacy on the connection between the supervisor–student relationship and academic procrastination is −0.082, the 95% confidence interval does not include 0, and the mediating effect is significant, supporting Hypothesis 2 (see Table 3). In the second path, the mediating effect of learning adaptation on the connection between the supervisor–student relationship and academic procrastination is −0.219, the 95% confidence interval does not include 0, and the mediating effect is significant, supporting Hypothesis 3 (see Table 3). In the third path, the chain-mediating effect of academic self-efficacy and learning adaptation on the connection between the supervisor–student relationship and academic procrastination is −0.112, the 95% confidence interval does not include 0, and the chain-mediating effect is significant, supporting Hypothesis 4 (see Table 3). This indicates that academic self-efficacy and learning adaptation act as chain-mediated mediators between the supervisor–student relationship and academic procrastination.

## 4. Discussion

### 4.1. The Influence of the Supervisor–Student Relationship on Academic Procrastination

This study found that the supervisor–student relationship has a significant negative effect on academic procrastination (*β* = −0.438, *p* < 0.001). That is, when graduate students have a good relationship with their supervisors, which is characterized by a high degree of trust, communication, and support, they are less inclined to procrastinate. The findings reinforce the view of leader–member exchange theory, which posits that if a subordinate maintains a high exchange relationship with his/her leaders, the subordinate will be regarded as an “insider” and will receive more support and resources from his/her leaders. In the relationship between the graduate student and the supervisor, the supervisor is equivalent to the leader, while the graduate student is equivalent to the subordinate. If the graduate student has a good supervisor–student relationship with the supervisor, they can get more resources and help from the supervisor. Graduate students who receive more support and resources from their supervisors are better placed to effectively deal with the challenges that arise in their academic pursuits. Furthermore, based on the principle of reciprocity of social exchange, graduate students who receive care and assistance from their supervisors will actively consider solutions, and strive to complete scientific research tasks on time for the purpose of reward their supervisors, thus reducing the occurrence of academic procrastination.

### 4.2. Academic Self-Efficacy and Learning Adaptation in the Relationship between the Supervisor–Student Relationship and Academic Procrastination

Our results show that the supervisor–student relationship can not only directly affect academic procrastination, but that this influencing process involves three mediating paths.

Firstly, the supervisor–student relationship can indirectly influence academic procrastination through the graduate student’s academic self-efficacy, wherein the higher the degree of self-efficacy, the lower the degree of academic procrastination. Social cognitive theory holds that the expectations, guidance, and social support of those who are important in one’s life will influence one’s levels of self-efficacy. In graduate studies, as the important person to graduate student, supervisors play a central role in guiding and developing the academic skills of their students, contributing to their students’ levels of academic self-efficacy. Our results are in line with previous studies which have also found that positive supervisor–student relationships promote students’ self-efficacy, while the reverse is true: negative supervisor–student relationships, such as abusive supervision, negatively affect students’ self-efficacy [29,49,50]. According to Bandura’s self-efficacy theory, the self-efficacy of individuals will have an impact on their behavior [18]. As a special kind of self-efficacy, academic self-efficacy will inevitably affect the behaviors related to research activities. Thus, when graduate students have high self-efficacy, they are better placed to deal with the problems that they encounter during their studies even if these problems prove challenging. This is because their confidence in their abilities leads them to seek solutions and to ensure that academic tasks are completed on time, however challenging they prove [19,22].

Secondly, the supervisor–student relationship can also indirectly affect academic procrastination through the mediating role of learning adaptation, where in the better the learning adaptation, the less inclined students are to procrastinate. The results of this study show that learning adaptation significantly predicts procrastination [39]. According to Bronfenbrenner’s developing ecology theory, the micro-environment and the meso-environment directly contacted by the individual have an important influence on the adaptation of the individuals [35]. As one factor of the meso-environment, the supervisor–student relationship can affect the adaptation of graduate students. When graduate students can adapt well to the new learning phase, which is more challenging and more demanding, they can devote their time and energy to completing the academic tasks at hand, rather than grappling with the challenges of maladjustment which contribute to procrastination.

Academic self-efficacy and learning adaptation can separately affect the relationship between academic procrastination and the supervisor–student relationship, but this study has also found that the mediating roles of academic self-efficacy and learning adaptation are linked like a chain. Social cognitive theory holds that good interpersonal relationships facilitate self-efficacy [24,25,26]. Graduate students who have good relationships with their supervisors are better placed to receive support and help from their supervisors, which in turn enhances the self-efficacy of these students. Bandura’s self-efficacy theory holds that the self-efficacy of individuals will have an impact on their behavior [18]. Individuals with high self-efficacy are better prepared psychologically to overcome difficulties and to actively adapt to changes in their environment. Previous studies have also found that academic self-efficacy significantly influences learning adaptation [51]. When graduate students can adapt well to their surroundings, their capacity to focus on the academic tasks at hand—however challenging these may prove to be—enables them to complete these tasks on time, rather than continually putting them off.

### 4.3. Limitations and Future Research Directions

There are several limitations of this study that should be noted. First, the cross-sectional study design is not enough to draw the conclusion of causality between supervisor–student relationship and academic procrastination. In the future, a follow-up study design or experimental design can be used to verify the conclusion of this study. Second, this study only considers the ways in which the supervisor–student relationship affects academic procrastination, but not the boundary condition of the influence itself. Future research can try to add moderation variables to discuss the boundary condition of the influence path. Third, we only recruited graduate students exclusively from Henan province. Because of this sampling, the findings of this study might not be generalizable to a population with higher educational attainment or to those in other geographical areas. Future research can try to use large samples to verify the conclusions of this study. Finally, the doctoral students were excluded in this study, and we did not pay attention to the differences between majors. Future research can try to consider these factors in research to verify the conclusions of this study.

## 5. Conclusions

(1) The supervisor–student relationship can directly predict the academic procrastination of graduate students, and there is a significant negative correlation between them.

(2) The relationship between graduate students and teachers can also indirectly and negatively predict graduate students’ academic procrastination through the mediating effects of academic self-efficacy and learning adaptation. Academic self-efficacy and learning adaptation can separately affect this relationship, but they can also have an influence on this relationship as linked mediators.

## Figures and Tables

**Table 1:** Means, standard deviations, and correlations (N = 818).

Variables	*M*	*SD*	1	2	3	4
1. Supervisor–student relationship	32.194	6.788	1			
2. Academic self-efficacy	13.335	3.140	0.627 **	1		
3. Learning adaptation	90.698	15.149	0.648 **	0.598 **	1	
4. Academic procrastination	9.460	3.410	−0.399 **	−0.424 **	−0.491 **	1

** *p* < 0.01.

**Table 2:** Results of the regression analyses (N = 818).

Variables	M1 Academic Procrastination		M2 Academic Procrastination	
	*β*	*t*	*β*	*t*
Supervisor–student relationship	−0.438	−12.464 ***	−0.026	−0.464
Academic self-efficacy			−0.125	−2.315 *
Learning adaptation			−0.449	−7.176 ***

* *p* < 0.05, *** *p* < 0.001.

**Table 3:** Results of chain-mediated effect test.

	Effect	Boot SE	Boot LLCI	Boot ULCI	Percentage
Indirect effect 1	−0.112	0.020	−0.157	−0.078	25.57%
Indirect effect 2	−0.082	0.036	−0.155	−0.011	18.72%
Indirect effect 3	−0.219	0.036	−0.300	−0.160	50.00%
Total indirect effect	−0.413	0.044	−0.203	−0.140	94.29%

## Data Availability

Data available on request due to restrictions privacy. The data presented in this study are available on request from the corresponding author. The data are not publicly available due to privacy.
